# The Toxicological Risk Assessment of Dermal Exposure of Patients Exposed to Nickel and Chromium due to Application of Ointments with Marjoram Herb Extract (*Majoranae Herbae Extractum*) Available in Polish Pharmacies

**DOI:** 10.1007/s12011-021-02798-9

**Published:** 2021-06-30

**Authors:** Kamil Jurowski, Maria Fołta, Barbara Tatar, Mehmet Berkoz, Mirosław Krośniak

**Affiliations:** 1 Institute of Medical Studies, Medical College, Rzeszów University, Al. mjr. W. Kopisto 2a, 35-959 Rzeszów, Poland; 2 Department of Food Chemistry and Nutrition, Medical College, Jagiellonian University, Medyczna 9, 30-688 Kraków, Poland; 3 Department of Biochemistry, Faculty of Pharmacy, Van Yuzuncu Yil University, 65080 Van, Turkey

**Keywords:** Nickel, Chromium, Toxicological risk assessment, Permitted daily exposure, Elemental impurities, Herbal medicinal products

## Abstract

For elemental impurities that have been studied for transcutaneous absorption, the available data are rarely suitable for proper toxicological risk assessment — there are multiple factors that can influence this dermal absorption. Hence, in our studies, we applied generic and conservative approach — cutaneous permitted daily exposure (CPDE) described in International Conference on Harmonisation’s Q3D Guideline on Elemental Impurities (ICH Q3D). The aim of this article is toxicological risk assessment (TRA) of dermal exposure of patients exposed to nickel and chromium due to application of ointments with Marjoram herb extract (*Majoranae herbae extractum*) available in Polish pharmacies (*n* = 5, because only five manufacturers produce this kind of pharmaceutical products in Poland). To make the appropriate TRA approach, we considered (1) raw results (metal per kg of ointment), (2) one-time administration of applied ointments, and (3) daily exposure *versus* CPDE. Due to the fact the concentrations of Ni generally present in cutaneous products as impurities are not considered sufficient to induce sensitization, the cutaneous and transcutaneous concentration limits (CTCLs) approach was applied for this element assessment. The toxicological analysis was carried out using microwave-assisted wet digestion with concentrated nitric acid and electrothermal atomization atomic absorption spectrometry. Our results show that the ointments with Marjoram herb extract from pharmacies in Poland represent a potential health hazard to patients; however, there may be a requirement for the monitoring of impurities of nickel in future. To the best of our knowledge, this paper is the first study about nickel and chromium content in ointments with Marjoram herb extract (*Majoranae herbae extractum*).

**Supplementary Information:**

The online version contains supplementary material available at 10.1007/s12011-021-02798-9.

## Introduction

**Fig. 1:**
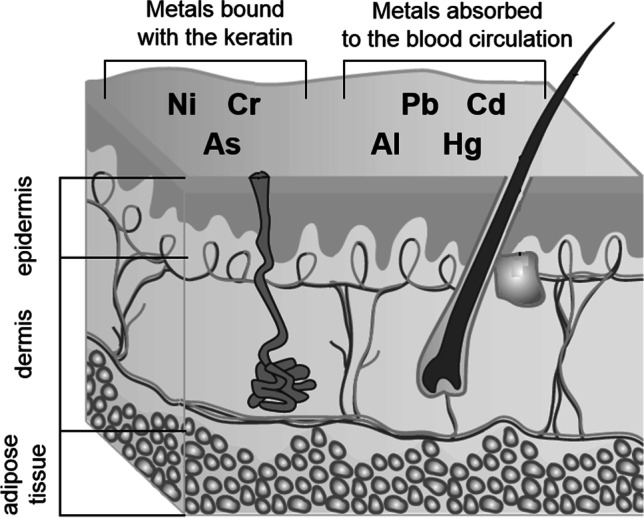
Elemental impurities accumulation (metals bound with the keratin) in the skin and absorption by the skin (metals absorbed to the blood circulation) — based on [2]

There is no doubt that role of the skin in establishing cutaneous and transcutaneous limits is extremely important [1]. Elemental impurities may be accumulated in the skin or absorbed by this route — Fig. 1.

**Fig. 2:**
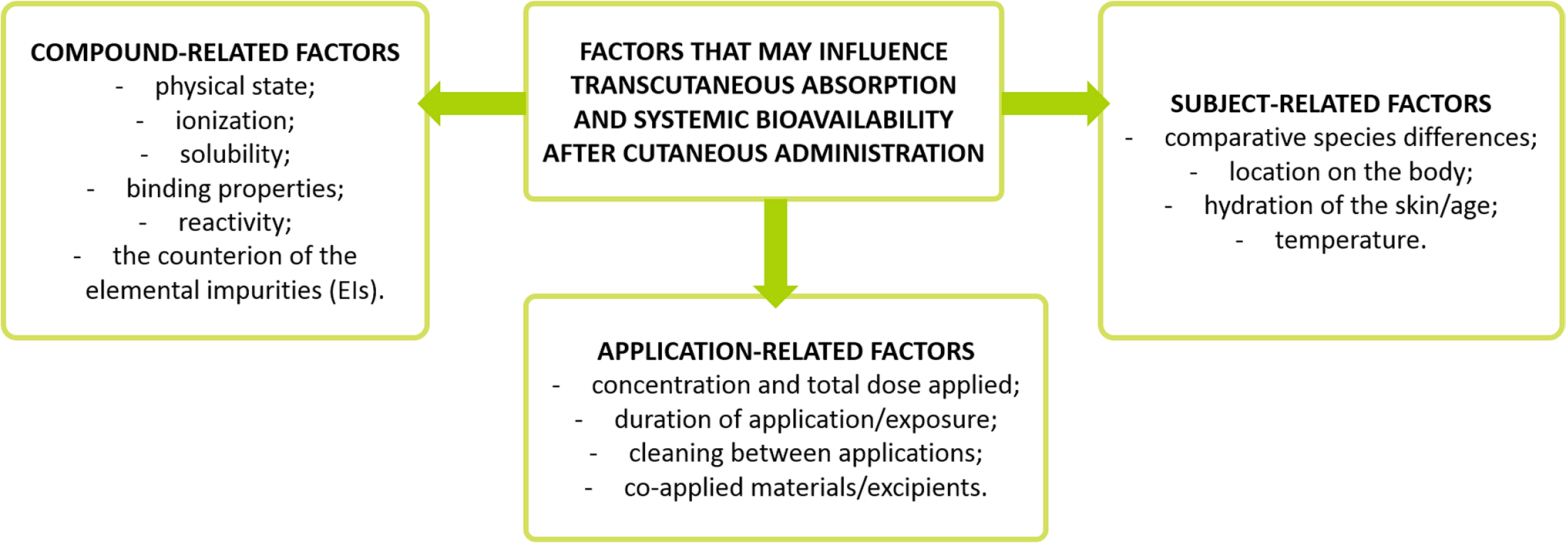
Factors that may influence transcutaneous absorption and systemic bioavailability after cutaneous administration of a substance — based on [1]

In classical safety assessment of substance applied in products for skin (especially safety assessment of cosmetic products), dermal/transcutaneous absorption is dependent upon the properties of the skin, the anatomical site, the nature of the chemical applied, and the characteristics of the application [3]. Moreover, there are numerous factors that may influence transcutaneous absorption and systemic bioavailability after cutaneous administration of a substance (Fig. 2).

It should be emphasized that there is a lack of studies about evaluation of the systemic absorption of elemental impurities (EIs) via the dermal route [3]. Published experimental data suggest that EIs are usually poorly absorbed through healthy skin even if enhancers are present [1, 3, 4]. Therefore, a conservative and generic strategy of risk characterization has been proposed for EIs by International Conference on Harmonisation’s Q3D Guideline [1]. This conservative, but very important, strategy takes into account application of the parenteral PDE (100% of bioavailability), to estimate a cutaneous permitted daily exposure (CPDE) using additionally appropriate correction factor (Cutaneous Modifying Factor, CMF). This risk characterization strategy has been estimated for daily/chronic application to the skin due to exposure for EIs in pharmaceutical products.

Based on literature review [5–7], the EIs in herbal medicine products are not a frequent subject of safety assessment. An interesting but downplayed and forgotten example of herbal medicine products with real and important source of EIs can be ointment with Marjoram herb extract (*Majoranae herbae extractum*) used adjunctively in rhinitis (runny nose). Marjoram herb extract has been known and used in traditional medicine for centuries, but nowadays is still used as a home remedy for runny nose (especially among young children and seniors) [8].

In scientific literature, there is a lot of articles focused on the determination of essential trace elements and toxic elements in many commercially available products [9–13]; however, there is a lack of studies about sensitizing metals (nickel) and problematic metals (chromium) in pharmaceutical herbal products, like ointment with Marjoram herb extract (*Majoranae herbae extractum*) used adjunctively in rhinitis (runny nose). To increase the knowledge around the exposure of the assessment of dermal exposure of patients exposed to nickel and chromium due to application of ointments with Marjoram herb extract, we have assessed the levels of these elements in ointments available in Polish pharmacies. In our study, we applied three approaches which are important from the toxicological risk assessment point of view: (1) raw results (metal per kg of ointment), (2) one-time administration of applied ointments, and (3) daily exposure.

For the reason that the concentrations of Ni generally present in cutaneous products as impurities are not considered sufficient to induce sensitization, the cutaneous and transcutaneous concentration limits (CTCLs) approach was applied for this element assessment. The assessment of dermal exposure of chromium was based on conservative CPDE approach.

## Materials and Methods

### Samples and Preparation

The samples were traditional herbal medicinal products used for relief of irritated skin around the nostrils. All pharmaceutical products (*n* = 5) were ointments with Marjoram herb extract (*Majoranae herbae extractum*) used adjunctively in rhinitis outfits (runny nose). All products were in semi-solid dosage forms (ointments) for cutaneous use. *Majoranae herbae extractum* consists of the dried flowering shoots of *Origanum majorana L*. containing not less than 5 mL/kg of essential oil (in the dried herbal substance). Usually, small amount of the ointment should be spread around nostrils, two to four times daily.

All herbal medicinal products were purchased from different pharmacies between March 2021 and April 2021 in Niepołomice and Kraków, Rzeszów, Poland. Ointments with Marjoram herb extract were chosen based on availability in Poland (only five manufacturers produce this kind of pharmaceutical products in Poland). To maintain the highest methodological standards, all samples were coded (as A, B, C, D, and E) before studies.

The short description of analyzed ointments tested in this study is described shortly in Table 1.

**Table 1:** Short description of analyzed samples

Sample		Main herbal component	Herbal preparation	Note
No	Code			
1	A	*Majoranae herbae extractum*	Extract (ratio of herbal substance to extraction solvent 1:5), extraction solvents: ethanol 96% V/V and white petroleum jelly	OTC
2	B			OTC
3	C			OTC
4	D			OTC
5	E			OTC

*OTC*, over-the-counter

Until analysis, each ointment was homogenized. Due to the fact all the ointment had an aluminum lid which could be a potential source of EIs, the first few centimeters of each ointment from the tube was discarded. Of each ointment, 0.25 g was measured, poured into teflon vessels, and digested with 5.0 mL of concentrated nitric acid (63%). The closed vessels left at room temperature for 2 h before microwave digestion. After this step, all samples were digested using microwave digestion system. Five replications were kept and done for all samples. The detailed information about digestion procedure and all instrumental parameters are described briefly in Supplementary Material 1. The samples were later cooled at room temperature (25 °C), and the final volume was made to 20 mL using ultrapure demineralized water. The cooled samples were stored in plastic bottles as stock sample solutions until analysis.

### Description of Toxicological Analysis

**Fig. 3:**
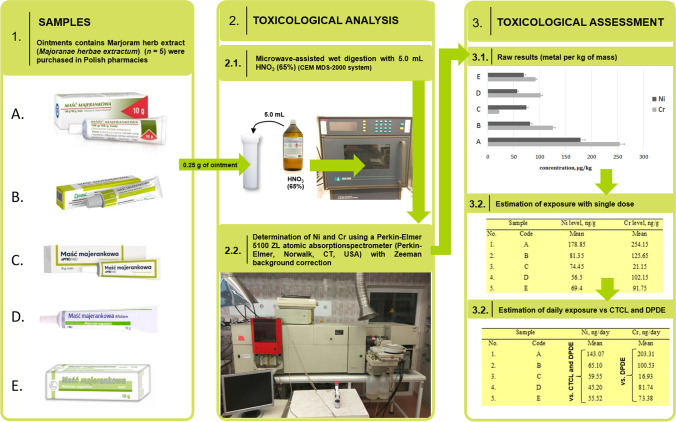
All steps in the assessment of dermal exposure of patients exposed to nickel and chromium due to application of ointments with Marjoram herb extract (*Majoranae herbae extractum*) available in Polish pharmacies

All steps of our research have been schematically summarized in Fig. 3.

 All instrumental parameters, and analytical calibration with quality control approaches are described briefly in Supplementary Material 1. Applied analytical approach was based on our studies published earlier [7, 10]. Five replications were performed for each sample. The quality control and validation of applied methodology are confirmed by previously described studies using the same methodology and apparatus [14].

Data were analyzed using statistical software Origin 2021 Pro The Ultimate Software for Graphing and Analysis (OriginLab Corporation, One Roundhouse Plaza, Suite 303, Northampton, MA 01,060, USA) licensed by the Jagiellonian University in Krakow. The resultant data of five independent replicates (five replicate samples from one tube of each preparation) were expressed as the mean ± standard deviation.

## Results and Discussion

### Ni and Cr Impurities Profile of Ointments with Marjoram Herb Extract (Majoranae Herbae Extractum) Available in Polish Pharmacies

**Fig. 4:**
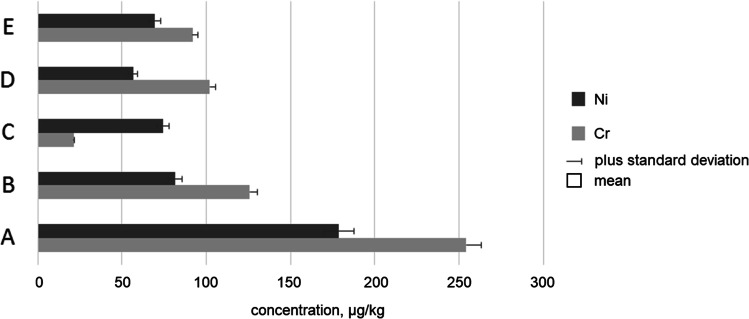
The Ni and Cr impurities profile of investigated herbal medical products samples (A, B, C, D, and E)

The impurities profile of investigated ointments with Marjoram herb extract (*Majoranae herbae extractum*) available in Polish pharmacies is shown in Fig. 4.

The highest levels of nickel and chromium were in sample A: 178.84 ± 6.85 μg/kg for Ni and 254.13 ± 12.59 μg/kg for chromium. The lowest content of Ni was in sample D (56.51 ± 3.22 μg/kg), and the lowest content of Cr was in sample C (21.16 ± 3.75 μg/kg). The nickel levels were similar for all herbal medicinal products (56.51–81.37 μg/kg), except sample A (178.84 ± 6.85 μg/kg — three times higher than lowest nickel level in other samples). On the other hand, the chromium level is variable (21.157–254.13 μg/kg). It can be assumed that in general, Ni levels were approximately similar (mean = 92.11 μg/kg) to Cr levels (mean = 118.97 μg/kg).

Very important issue about nickel sensitization potential is the fact that the content of this element present in product applied by dermal route is not clearly correlated sufficiently with the induction of sensitization [1]. Hence, in this situation (similar to Co), the consideration of appropriate concentration limit as the cutaneous and transcutaneous concentration limit (CTCL) [1] is required. The CTCL value for nickel was established by European Medicines Agency (EMA) as 35 μg/kg [1]. Analysis of the obtained results shows that CTCL value was exceeded in all investigated samples. There is no doubt that each product poses a potential allergic risk due to Ni content. For Cr assessment, additional approaches due to sensitization potential are not required based on ICH Q3D recommendations [1] and Nethercott et al. article [15].

### Level of Nickel and Chromium Impurities Including Single Dose of Ointments with Marjoram Herb Extract (*Majoranae Herbae Extractum*)

Obtained results of nickel and chromium levels in the ointments with Marjoram herb extract (*Majoranae herbae extractum*) available in Polish pharmacies are potentially important for other scientist and regulatory affair specialist in pharmaceutical industry (comparative analysis). However, appropriate toxicological risk assessment of investigated metallic elements in this kind of pharmaceuticals should include more information. Required information in this situation is actual level in the one-time administration (single dose) of the product (approximately 0.20 g). The levels of nickel and chromium in analyzed samples (ointment, ng/g) are presented in Table 2.

**Table 2:** The levels of nickel and chromium in analyzed samples (ointment, ng/g)

Sample		Ni level, ng/g		Cr level, ng/g	
No	Code	Mean	SD	Mean	SD
1	A	178.85	8.22	254.15	12.25
2	B	81.35	5.78	125.65	7.85
3	C	74.45	3.25	21.15	1.59
4	D	56.5	2.31	102.15	6.45
5	E	69.4	3.44	91.75	5.44

*SD* standard deviation

This step of calculation is indispensable for the next level of toxicological risk assessment — the daily dermal exposure of investigated elements (the maximum daily dose of applied pharmaceuticals).

### Estimation of Daily Exposure of Ni and Cr Versus Dermal Permitted Daily Exposure (DPDE)

Based on information in the leaflet for each ointment and information from assessment report on *Origanum majorana* L. from EMA [8], small amount of the ointment should be spread around nostrils, two to four times daily. The estimated daily exposures to nickel and chromium through applied ointments were calculated considering the maximum use during the day — Table 3. We use five applications as recommended maximum application to calculate the results from Table 3.

**Table 3:** The estimated daily exposure of investigated metals in analyzed ointment (ng/day)

Sample		Ni, ng/day		Cr, ng/day	
No	Code	Mean	SD	Mean	SD
1	A	143.07	8.22	203.31	12.25
2	B	65.10	5.78	100.53	7.85
3	C	59.55	3.25	16.93	1.59
4	D	45.20	2.31	81.74	6.45
5	E	55.52	3.44	73.38	5.44

*SD* standard deviation

Results from Table 3 show that the estimated exposure of Ni levels is variable (45.20–143.07 ng/day). Additionally, the estimated exposure of Cr is also variable (16.93–203.31 ng/day). Considering dermal route and cutaneous bioavailability (< 1% [1]), cutaneous PDE should be applied in toxicological risk assessment. As was mentioned in “Introduction,” a generic and conservative approach has been applied for EIs by International Conference on Harmonisation’s Q3D Guideline on EIs based on a systematic adjustment of the parenteral PDE, which assumed 100% bioavailability, to derive a cutaneous permitted daily exposure by using a Cutaneous Modifying Factor (in most cases — intact/irritated skin 10; 100%/10% = 10) — Eq. 1.

1 $$\mathrm{C}\mathrm{P}\mathrm{D}\mathrm{E}=\mathrm{P}\mathrm{a}\mathrm{r}\mathrm{e}\mathrm{n}\mathrm{t}\mathrm{e}\mathrm{r}\mathrm{a}\mathrm{l}\mathrm{P}\mathrm{D}\mathrm{E}\times \mathrm{C}\mathrm{M}\mathrm{F}$$

where

CPDE — cutaneous permitted daily exposure;

Parenteral PDE — parenteral permitted daily exposure;

CMF — Cutaneous Modifying Factor.

The calculations of CPDE for Ni and Cr based on [1] are shown in Table 4.

**Table 4:** The calculations of cutaneous permitted daily exposure (CPDE) for Ni and Cr

	Ni	Cr
Parenteral PDE (µg/day)	20	1100
CMF	10	10
CPDE (µg/day)	200	11,000

Acronyms: *PDE*, permitted daily exposure; *CMF*, Cutaneous Modifying Factor; *CPDE*, cutaneous permitted daily exposure

This simple, conservative but very useful approach has been estimated for daily/chronic application of pharmaceuticals via dermal route including EIs assessment [1]. The applied toxicological risk assessment approach confirms that the estimated dermal Ni daily exposure (μg/day) is below the CPDE value for this element (< 200 μg/day) in all investigated herbal-based pharmaceutical products. It should be recalled that the full assessment of this element should also include allergenic potential [1], what was described in impurities profile section (see earlier). It can be summarized that level of nickel in all investigated medicaments with Marjoram herb extract exceeds the CTCL (> 35 μg/kg, see Fig. 3 and Fig. 4).

On the other hand, the estimated daily exposure of Cr is significantly variable (16.93–203.31 ng/day). The established by EMA CPDE for this element is 11000 μg/day. It can be assumed that all analyzed samples in our studies are characterized by levels of Cr below the established CPDE. Hence, the applied toxicological risk assessment confirms safety of investigated herbal medicinal product with Marjoram herb extract due to estimated daily exposure of this element.

## Conclusions and Recommendations

The levels of Ni and Cr as EIs in all ointments with Marjoram herb extract (*Majoranae herbae extractum*) available in Polish pharmacies are very low. However, the concentrations of EI generally present in cutaneous products as impurities are not considered sufficient to induce sensitization (especially considering Ni) [1–3]. From this point of view, additional assessment including CTCL was done; level of nickel in all investigated herbal medicinal products exceeds the CTCL (> 35 μg/kg). This indicates a potential allergy associated with the presence of nickel in the ointments.

Considering the level of nickel and chromium in single dose of applied ointment, again, is also at a very low level. This simple, conservative but very useful risk characterization strategy has been estimated for daily/chronic dermal route. The toxicological risk assessment approach confirms that the estimated dermal daily exposure of investigated metals in comparison to the CPDE in all products is below established EMA requirements. Hence, the obtained results are in accordance with the standards of directive ICH Q3D.

It can be assumed that analyzed products with Marjoram herb extract in our study represent a health hazard for patients. It should be emphasized that there is a potential risk of nickel allergy (all samples exceed the CTCL). For this reason, a monitoring nickel content in herbal-based pharmaceuticals available in European Union will be desirable. Moreover, herbal medicinal products should be monitored for the presence of other important and potentially hazardous EIs [16–18]. Finally, our results are important and may be significant for other scientist and regulatory affairs working in pharmaceutical industry, especially due to PDE-related topics.

## Supplementary Information

Below is the link to the electronic supplementary material.

ESM 1 (DOCX 20 kb)

## Data Availability

All data generated or analyzed during this study are included in this published article and its supplementary information file.

## Abbreviations

CPDE: Cutaneous permitted daily exposure
CTCLs: Cutaneous and transcutaneous concentration limits
EMA: European Medicines Agency
EIs: Elemental impurities
ET AAS: Electrothermal atomization atomic absorption spectrometry
ICH Q3D: International Council for Harmonization of Technical Requirements for Pharmaceuticals for Human Use
PDE: Permitted daily exposure
SD: Standard deviation
TRA: Toxicological risk assessment
