# Supplementary material for: The Toxicological Risk Assessment of Dermal Exposure of Patients Exposed to Nickel and Chromium due to Application of Ointments with Marjoram Herb Extract (Majoranae Herbae Extractum) Available in Polish Pharmacies
Source: Biol Trace Elem Res. 2021 Jun 30;200(4):1965–71. doi: 10.1007/s12011-021-02798-9 (PMC8854252; doi:10.1007/s12011-021-02798-9)
Supplement: Supplementary file 1 — (DOCX 20 kb) [file 12011_2021_2798_MOESM1_ESM.docx]

**Supplementary Material 1.**

*1. Samples preparation procedure*

The samples were digested using microwave digestion system (CEM, Matthews, NC, USA). Concentrated nitric acid (65%) for microwave digestion were of spectroscopic grade from Merck (Darmstadt, Germany). The A microwave-assisted digestion procedure is shown briefly in table S1.

**Table S1.** A microwave-assisted digestion procedure.

|  | Step 1 | Step 2 | Step 3 | Step 4 | Step 5 |
| --- | --- | --- | --- | --- | --- |
| Power, W | 80 | 100 | 100 | 0 | 0 |
| PSI | 80 | 100 | 150 | 20 | 20 |
| Time, minutes | 6:00 | 6:30 | 7:00 | 5:00 | 5:00 |
| TAP, minutes | 2:00 | 5:00 | 5:00 | 0 | 0 |
| Fan power, % | 100 | 100 | 100 | 100 | 100 |

Abbreviations: PSI – pounds-force per square inch; TAP – time at pressure min.

*2. Chemicals and Reagents*

All solutions were prepared with ultrapure demineralised water that had been obtained by Milli-Q water purification system (Millipore, Bedford, MA, USA). Purge gas was argon at purity 99.99%. The certified reference material (Corn Flour, INCT-CF-3) was purchased from the Institute of Nuclear Chemistry and Technology - Department of Analytical Chemistry (Warsaw, Poland). Working solutions of nickel and chromium were prepared from the stock solutions of 1000 μg/mL (1 mg/mL) using ultrapure demineralised water in 0.5 mol/L nitric acid: Ni(NO_3_)_2_ and Cr(NO_3_)_3_. Five working solutions with a concentration of 0.0; 12.5; 20.0; 50.0 and 100.0 µg/L were prepared and used for the analytical calibration.

*3. Determination of nickel and chromium*

The determination of Ni and Cr in the pharmaceutical samples was carried out using a Perkin-Elmer 5100 ZL atomic absorption spectrometer (Perkin-Elmer, Norwalk, CT, USA) with Zeeman background correction and with electrothermal atomization (ET AAS technique). The time-temperature program in the graphite furnace atomic absorption spectrometer for nickel and chromium determination is detailed described in table S2.

**Table S2.** Time-temperature program in the graphite furnace atomic absorption spectrometer for Ni and Cr determination

| Ni determination | Step | Temperature, °C | Ramp, s | Hold, s | Gas Flow, mL/min |
| --- | --- | --- | --- | --- | --- |
|  | 1 | 110 | 1 | 50 | 250 |
|  | 2 | 700 | 10 | 20 | 250 |
|  | 3 | 2350 | 0 | 5 | 0/read |
|  | 4 | 2400 | 1 | 3 | 250 |
|  | 5 | 110 | 1 | 10 | 250 |
| Cr determination | Step | Temperature, °C | Ramp, s | Hold, s | Gas Flow, mL/min |
|  | 1 | 110 | 5 | 15 | 250 |
|  | 2 | 180 | 30 | 10 | 250 |
|  | 3 | 450 | 1 | 5 | 250 |
|  | 4 | 500 | 5 | 5 | 250 |
|  | 5 | 1500 | 15 | 30 | 250 |
|  | 6 | 2450 | 0 | 5 | 0/read |
|  | 7 | 2500 | 1 | 2 | 250 |

The emission source were hollow-cathode lamps for nickel (228.8 nm, 5 mA) and chromium (357.9 nm, 8 mA). Analytical quality control was performed using certified reference material (Corn Flour, INCT-CF-3): for Ni: 0.383 mg/kg certified value, and 0.386 mg/kg measured value; for Cr: 0.137 mg/kg certified value, and 0.134 mg/kg measured value. The recoveries were 100.8% and 98% for Ni and Cr respectively. The recoveries were calculated as the quotient of the determined level and the known amount of the determined element expressed as a percentage. The LODs for the metals were 1.93 μg/L for Ni, and 1.65 μg/L for Cr. The LOQs for the metals were 5.79 μg/L for Ni, and 4.95 μg/L for Cr. Calibration functions for all metals indicated good correlation coefficients (R) greater than 0.998 (R_Ni_ = 0.9985;
R_Cr_ = 0.9991). This implies that there was good linearity of instrumental response with metal concentrations. Five replications were performed for each sample. Data were analysed using Educational Analysis Set SAS® 9 licenced by the Jagiellonian University in Krakow. The mean and RSD were calculated.
